# Online Lectures in Undergraduate Medical Education: Scoping Review

**DOI:** 10.2196/mededu.9091

**Published:** 2018-04-10

**Authors:** Brandon Tang, Alon Coret, Aatif Qureshi, Henry Barron, Ana Patricia Ayala, Marcus Law

**Affiliations:** ^1^ Faculty of Medicine University of Toronto Toronto, ON Canada; ^2^ Gerstein Science Information Centre University of Toronto Toronto, ON Canada

**Keywords:** online lectures, undergraduate medical education, multimedia design, assessment, scoping review, e-learning

## Abstract

**Background:**

The adoption of the flipped classroom in undergraduate medical education calls on students to learn from various self-paced tools—including online lectures—before attending in-class sessions. Hence, the design of online lectures merits special attention, given that applying multimedia design principles has been shown to enhance learning outcomes.

**Objective:**

The aim of this study was to understand how online lectures have been integrated into medical school curricula, and whether published literature employs well-accepted principles of multimedia design.

**Methods:**

This scoping review followed the methodology outlined by Arksey and O'Malley (2005). Databases, including MEDLINE, PsycINFO, Education Source, FRANCIS, ERIC, and ProQuest, were searched to find articles from 2006 to 2016 related to online lecture use in undergraduate medical education.

**Results:**

In total, 45 articles met our inclusion criteria. Online lectures were used in preclinical and clinical years, covering basic sciences, clinical medicine, and clinical skills. The use of multimedia design principles was seldom reported. Almost all studies described high student satisfaction and improvement on knowledge tests following online lecture use.

**Conclusions:**

Integration of online lectures into undergraduate medical education is well-received by students and appears to improve learning outcomes. Future studies should apply established multimedia design principles to the development of online lectures to maximize their educational potential.

## Introduction

The modern classroom has changed significantly since the days of paper and pencil learning. Increasing numbers of elementary and secondary school students are using online textbooks, writing their tests online, and watching videos created by their teachers [1]. Accordingly, medical students who have grown up in this digital age are currently experiencing one of the most significant transformations in medical education [2]. In particular, the adoption of the flipped classroom model is reshaping undergraduate medical education by calling on students to learn from a variety of self-paced tools—including online lectures—before attending live teaching sessions [3]. This allows class time with instructors and peers to focus on a discussion of applications, clinical context, and more nuanced or challenging topics. Thus, the design of online lectures merits special attention as they become a more widespread teaching modality for foundational medical concepts.

**Summary of multimedia design principles with definitions reproduced from the Association of American Medical Colleges Institute for Improving Medical Education's *Effective Use of Educational Technology in Medical Education*.**

- Coherence: exclude extraneous words, pictures, and sounds
- Pretraining: ensure students possess prior knowledge about names and characteristics of the main concepts
- Spatial contiguity: present corresponding words and pictures in close proximity to one another
- Temporal contiguity: present corresponding words and pictures simultaneously rather than successively
- Signaling: highlight important words
- Redundancy: pair animation and narration together without on-screen text
- Voice: use non-accented human spoken voice for narration over a machine simulated or foreign-accented human voice
- Personalization: employ conversational style, instead of formal, to present words
- Segmenting: offer narrated animation in learner-paced segments rather than a continuous unit
- Modality: pair animation and narration together instead of pairing animation and on-screen text

For the purposes of this review, online lectures were defined as primarily didactic lectures accessed through digital platforms that do not require active interaction with the video playback interface. Examples of interactivity which would merit exclusion as an online lecture included instructional media in which students “click through” or complete “drag and drop” activities.

In 2007, the Association of American Medical Colleges Institute for Improving Medical Education (AAMC-IIME) published the landmark report *Effective Use of Educational Technology in Medical Education*. One principal recommendation was that medical educators employ established multimedia design principles when developing instructional materials. These design principles initially emerged from educational psychology literature, as described by Richard Mayer’s * Theory of Multimedia Learning*. Mayer described empirical evidence that people learn more effectively from multimedia, or words and pictures together, than words alone. However, simply adding words to pictures is not effective, as instructional media must be designed in accordance with how the human mind works. Based on his empirical research, Mayer outlined multimedia design principles as guiding concepts to enhance learning from multimedia presentations [4]. These principles include pragmatic concepts such as removing extraneous words, employing a conversational style, and reducing redundancy across animation, narration, and on-screen text [5]. A comprehensive list of multimedia design principles can be found in Textbox 1. In the context of medical education, it has been shown that applying multimedia design principles to medical student lectures leads to improved attainment of learning objectives both immediately and long-term [6,7].

Despite the purported benefit of careful multimedia design, it is unclear whether best practice has become routine practice in medical education. The AAMC-IIME note that a cultural lag often occurs between the development of novel educational technologies and their effective implementation [5]. Therefore, the purpose of this scoping review was to understand how online lectures have been integrated into medical school curricula, and whether multimedia design principles are being utilized in their creation. With the emergence of online lectures as an increasingly prevalent teaching modality—and given the significant resources being allocated to their development—understanding the application of best practice in online lecture design is of significant and immediate relevance.

## Methods

We searched OVID Medline (1946 to present, In Process & Other Non-Indexed Citations), OVID PsycINFO (1806 to present), OVID Social Work Abstracts (1968 to present), EBSCO Education Source, PROQUEST Abstracts in New Technology & Engineering, ASSIA, Canadian Research Index, CBCA Education, Computer & Information Systems Abstracts, ERIC, Computer Science Collection, Engineering Journals and PSYCTESTS, and FRANCIS, to identify articles addressing the subjects of online learning and medical education. Search strategies were developed by an academic health science librarian (APA) with input from the project leads and content experts (ML, BT). The search strategies were translated using each database platform’s command language, controlled vocabulary, and appropriate search fields. Medical Subject Headings terms, American Psychological Association thesaurus terms, and text words were used for the search concepts of “e-learning”, “video lectures”, “medical education”, and “medical students”. Searches were completed on July 1, 2016 and limited to articles published between July 1, 2006 and July 1, 2016, given that we were predominantly interested in examining literature published since the release of the AAMC-IIME report in March 2007. English-language limits were applied to all databases.

All articles were independently screened (by 2 of BT, AC, AQ, or HB) through a 2-step process of abstract and full-text review to determine eligibility for inclusion. Only articles that were not excluded through abstract review underwent full-text review. Articles that ultimately met inclusion criteria were then analyzed and charted according to the following iteratively developed categories: (1) lecture topic; (2) participants and setting; (3) lecture design components; (4) process of lecture design; (5) method of assessment; and (6) results.

Primary research articles written in English were included if they (1) discussed online, didactic lectures whose primary purpose was to teach or review curricular content; (2) did not require active interaction with the video playback interface; (3) were created by or for a medical school; (4) involved undergraduate medical students; (5) were watched independently by students; and (6) included either video, a slide deck, or an informal talking head. Articles were excluded if they discussed teaching modalities that required active participation (eg, problem-based learning), were not online, involved nonmedical doctor health care students, involved advanced trainees (eg, medical residents), were not designed by or for the medical school (eg, external YouTube channel), were watched by students in a group setting, or involved a lecture that was not a core educational component (eg, used for an extracurricular activity).

Assessment methods were then categorized according to the Kirkpatrick 4-level model of evaluation, interpreted in the context of online lecture evaluation [8]. Level 1 (reaction) was defined as learner satisfaction or confidence; Level 2 (learning) was defined as knowledge of information directly taught in the online lecture; Level 3 (transfer of learning) was defined as improved outcomes in tasks not directly taught in the online lecture (eg, practical examinations or final course grades); and Level 4 was defined as benefit to patients or organizational practice (eg, improved clinical outcomes such as quality of care).

Lastly, the rigor of studies included in the final analysis was assessed using the Newcastle-Ottawa Scale (NOS) [9]. The NOS is a widely used scale with established content validity and inter-rater reliability. It judges studies based on the following key parameters: (1) the selection of the study groups; (2) the comparability of the groups; and (3) the outcome measures employed. Within the context of this work, 2 of the authors (BT and AC) coded the articles included in the final analysis (N=45) according to the criteria outlined in the NOS. Any disagreements were resolved via a consensus discussion, and remaining areas of ambiguity were deliberated with other members of the research team.

## Results

**Figure 1:**
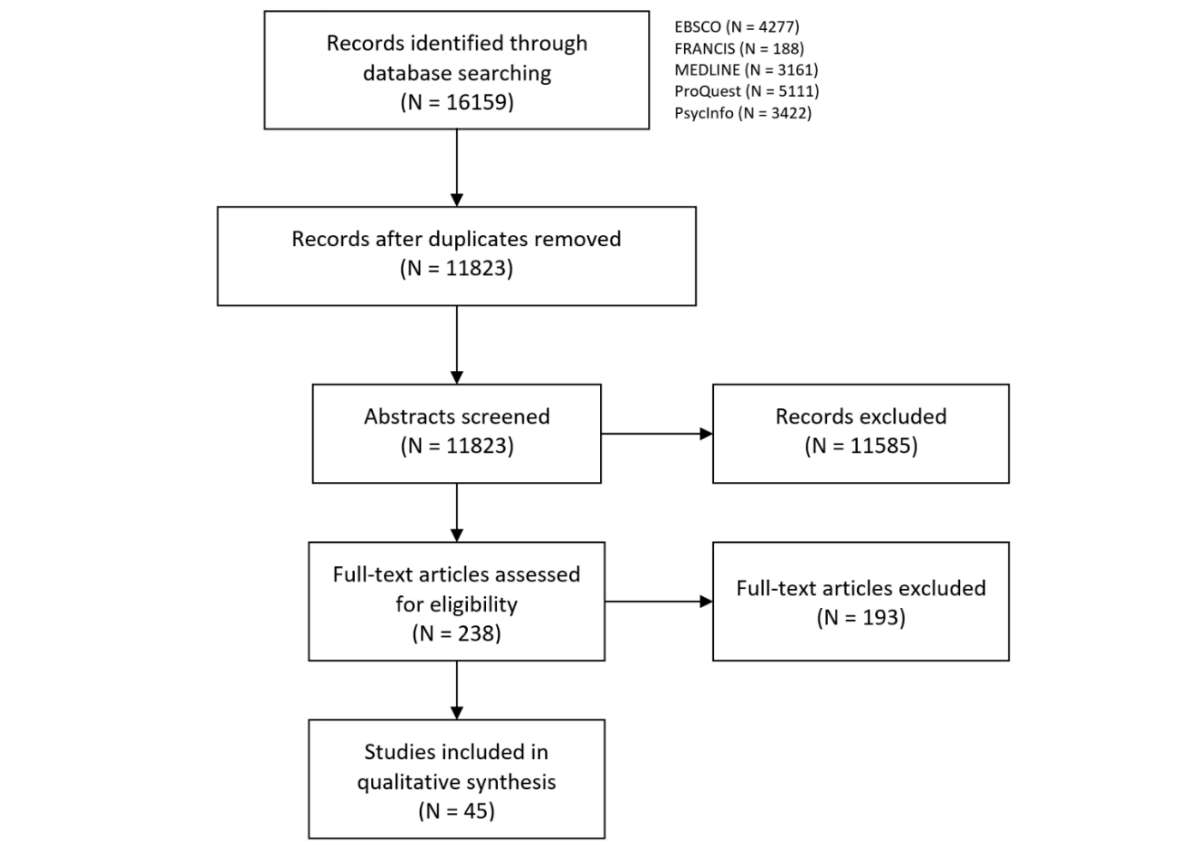
Preferred Reporting Items for Systematic Reviews and Meta-Analyses (PRISMA) flow chart for the article search.

Our search revealed 16,159 potentially relevant studies, of which 45 articles ultimately met inclusion criteria (Figure 1). Of the 238 articles that underwent full-text review, 193 (193/238, 81.1%) were excluded because they involved nondidactic lectures (75/193, 38.9%), were not primary research (51/193, 26.4%), involved nonmedical student populations (25/193, 13.0%), were duplicate articles identified through different search databases (24/193, 12.4%), had no involvement of online lectures (16/193, 8.3%), or involved videos that were not designed by the medical school (2/193, 1.0%).

### Lecture Topics, Participants, and Setting

Online lectures were employed in preclinical and clinical years, covering diverse topics such as basic sciences (12/45, 27%), clinical medicine (16/45, 36%), and clinical skills (17/45, 38%). Please refer to Multimedia Appendix 1 for a summary of all included studies, tabulated by lecture topic(s), participants and setting, lecture component(s), lecture design process, assessment method(s), assessment Kirkpatrick Level(s), and summary of results.

### Lecture Components and Design Processes

The most common elements of online lectures included slide decks (25/45, 56%), narration (23/45, 51%), and video (18/45, 40%), with slide decks and narration typically occurring in conjunction. Several studies used the terminology online “lecture” or “module”, but did not clarify the specific design of these interventions (5/45, 11%). A summary of design features can be found in Table 1. Approaches to delivering online lectures were occasionally described as well, with 16% (7/45) of lectures reported as case-based, 13% (6/45) of lectures including self-assessment questions, and 11% (5/45) of lectures including links to additional resources.

Of the studies, 56% (25/45) commented on the development of online lectures in terms of process, content, or design (Table 2). The most frequently described process of lecture design included partnership with medical students (6/45, 13%), and either redesigning existing live lectures for an online platform or uploading recordings of live lectures onto an online portal (10/45, 22%). Only 3 studies (3/45, 7%) commented on the use of multimedia design principles, such as the purposeful design of slide topography to enhance student learning [10-12]. Lecture content was typically selected based on existing curriculum objectives or according to expert recommendations from national organizations (7/45, 16%), such as the 6-step approach to curriculum development developed by Kern et al [13].

### Methods of Assessment

All studies assessed learning outcomes (Table 3), with the most common method (39/45, 87%) being self-assessment of satisfaction, knowledge acquisition, or confidence. These all represent Kirkpatrick Level 1 and involved surveys, questionnaires, or focus groups for evaluation purposes. Higher-order assessment (Kirkpatrick Level 2) included various knowledge tests such as multiple choice, true/false, matching, key feature, or free response questions (30/45, 66%). Of all studies, 18% (8/45) assessed learning through objective structured clinical examinations (OSCEs) or other practical examinations, while 24% (11/45) correlated the use of online lectures with other performance measures, such as final course grades or *United States Medical Licensing Examination* (USMLE) scores. Both practical examinations and correlation to other external measures (eg, USMLE) were typically defined as Kirkpatrick Level 3, given that knowledge from the online lectures was being applied to new contexts beyond the content directly addressed in the lecture.

**Table 1:** Summary of online lecture design components (N=45).

Design component^a^	n (%)
Slide deck (eg, Microsoft PowerPoint)	25 (56)
Audio or narration	23 (51)
Video (eg, procedural demonstration; does not include video recordings of slide decks)	18 (40)
Unspecified design (eg, only described as online “lecture” or “module”)	5 (11)
Animation (eg, dynamic 2D or 3D images)	4 (9)
Visible lecturer (eg, talking head)	1 (2)

^a^Articles often utilized more than one design component.

**Table 2:** Summary of online lecture development (N=45).

Development process^a^		n (%)
**Lecture design**			
	No comment on development process	20 (44)
	Developed from live lectures or recordings of live lectures	10 (22)
	Medical student consultation	6 (13)
	Consideration of multimedia design principles (eg, slide topography)	3 (7)
**Lecture content**			
	Literature-driven development of content (eg, 6-step approach to curriculum development from Kern et al [13] or national specialty-specific guidelines or learning objectives)	7 (16)
	Faculty or expert selection of content	6 (13)

^a^Articles often utilized more than one development process.

**Table 3:** Summary of assessment methods for online lectures (N=45). OSCE: objective structured clinical examination; USMLE: *United States Medical Licensing Examination*.

Assessment method^a^	Kirkpatrick Level	n (%)
Any method	1 to 3	45 (100)
Self-assessment of satisfaction, attitudes, knowledge, or confidence (eg, survey, questionnaire, or focus group)	1	39 (87)
Knowledge assessment (eg, multiple choice, true/false, matching, key feature, or free-response questions; content-specific knowledge test such as electrocardiogram interpretation)	2	30 (66)
Correlation to other performance measures (eg, final course grades, or USMLE)	3	11 (24)
Practical assessment (eg, OSCE, practical examination, or direct observation)	3	8 (18)
Written assignment or project	2	4 (9)

^a^Articles often utilized more than one assessment method.

In some cases, a practical examination was defined as Kirkpatrick Level 2, when the examination tested direct transfer of knowledge from the lecture (eg, lecture on differential diagnosis generation and an oral examination on the same topic).

Regardless of the method of assessment, almost all studies reported high satisfaction and increased knowledge following the intervention. Student self-assessment typically revealed positive attitudes toward online lectures as a teaching modality [10,11,14-31]. Moreover, students showed increased knowledge in the subject material at hand, as evaluated by pre- and postlecture knowledge assessment [14,16-18,20,22,24,25,27,28, 30,32-35]. Finally, multiple studies demonstrated that knowledge was equivalent (or better) between students learning through online lectures compared to traditional learning modalities, such as live didactic lectures [11,12,15,22,25,26,28,31,36-41], with the exception of one study that found superior student knowledge acquisition from live lectures [42].

The quality and rigor of studies included in the final analysis (N=45) were evaluated based on the criteria set out by the NOS [9]. Out of 45 studies, only 21 (21/45, 47%) had clearly established “control” and “intervention” groups, whereas the remaining studies examined learning outcomes by following a specific cohort of students over time. Moreover, studies that randomized students into 2 (or more) specific groups had, on average, more participants (210 versus 168). With regards to outcomes, the majority of studies (37/45, 82%) utilized some form of blind—or at least, objective—assessment of learning, most commonly via test performance (eg, USMLE, pre- versus postintervention testing, OSCEs, etc). The remaining minority of studies (8/45, 18%) relied solely on student satisfaction ratings of the online media.

## Discussion

### Integration of Online Lectures into Medical Curricula

This review demonstrated that online lectures have been integrated into several aspects of undergraduate medical education curricula, tailored toward diverse subject matter and learners at all levels. This suggests that the flipped-classroom model—and associated online lectures—have become widely embraced by medical educators. Although preclinical students appear to prefer live lectures when given the option, online lectures are perceived to allow for increased rate and quantity of knowledge acquisition [43]. Online lectures may also be valuable for students in clinical settings, given the time constraints on preceptors to simultaneously teach and tend to their clinical responsibilities [44].

### Online Lecture Design

Results from this review demonstrated that 10 years after the publication of *Effective Use of Educational Technology in Medical Education* [5], there has been a cultural lag in implementing multimedia design principles. As stated earlier, emerging evidence suggests that applying multimedia design to medical student lectures can improve learning outcomes [6,7]. Moreover, since the publication of the AAMC-IIME report in 2007, the importance of applying multimedia design principles in medical education has been emphasized in multiple publications [45,46]. Previously described barriers to implementing best practice in clinical medicine may explain the cultural lag in applying multimedia design principles in medical education, including time constraints (organizational context) and existing standards of practice (social context) [47]. Multiple studies included in this review implemented online lectures as part of broader educational interventions, and therefore, lack of time or resources may have reduced the attention paid to online lecture design. Moreover, clinician-teachers who participate in online lecture design may be unaware of multimedia design principles or may not have integrated these concepts into their standard practice. In line with this, findings from this review suggest an overall lack of awareness of the importance of conscientious online lecture design in the medical education community. Almost half of all included articles did not comment on the development process for online lectures, while nearly a quarter of studies simply uploaded lecture recordings online or repurposed slide decks from live lectures into online lectures.

### Assessment of Learning Outcomes

The most common method of assessment involved student self-assessment (Kirkpatrick Level 1), consistent with other reports of assessment in medical education [48]. However, several studies did examine learning outcomes in a more objective way (eg, written test or OSCE), with the general trend being one of noninferiority for students participating in an online and/or blended educational intervention. Nonetheless, it is also important to note that in some studies, online lectures represented only one aspect of a broader curricular intervention (eg, a new program to teach bedside ultrasound). Therefore, the impact of online lectures cannot always be delineated from other aspects of an intervention, especially with respect to complex outcome measures that integrate multiple knowledge domains.

Essentially all studies reported high student satisfaction with online lectures and improved knowledge following such an intervention. However, each study assessed the use of online lectures within a particular context of students, educational topics, and assessment methods, making it difficult to directly compare relative effectiveness. Nonetheless, the broader body of literature suggested that online lectures, as a whole, were widely applicable and effective. Although positive outcomes were almost uniformly described, multimedia design principles were employed in only 3 studies, suggesting that these interventions could further optimize student learning by applying these well-established concepts [10-12].

Multiple studies reported equivalent or superior learning outcomes in medical students learning from online lectures compared to traditional didactic teaching. These findings are consistent with a large meta-analysis conducted by the United States Department of Education, which found that kindergarten to grade 12 (K-12) students in online learning conditions had better learning outcomes than those receiving in-person instruction [49]. However, the authors of this study cautioned that this does not necessarily suggest that online learning is the superior medium. Rather, it may be the conditions associated with online lectures (eg, additional learning time or access to extra resources), that lead to improved learning outcomes.

### Towards More Effective Use of Online Lectures

Online teaching modalities included didactic online lectures (the definition employed in this review), interactive online modules, online courses, and many other interventions. However, the term “online lecture” was used to refer to a diverse range of online teaching modalities in published studies, and sometimes without an accompanying description of the lecture. Applying common terminology when describing online teaching modalities would help medical educators communicate more clearly about the nature of interventions, as well as delineate between different intervention designs to facilitate the study of their relative effectiveness. 

In line with this goal, we propose standardized definitions to describe different online teaching modalities (Textbox 2). Accordingly, precise documentation of design processes for these different modalities can counter the cultural lag described and better disseminate an approach to transitioning toward “flipped classroom” undergraduate medical education curricula.

Further research would be helpful to identify the specific design features of online lectures that best facilitate medical student learning, given the widespread but variable application of this teaching modality. For example, future research could investigate which multimedia design principles correlate best with improved learning outcomes. Moreover, an understanding of the effectiveness of online lectures (didactic) compared to online modules (interactive), and the settings in which each modality is best applied, would allow for more purposeful application of online teaching interventions. Finally, to bridge the gap between effective use and common practice, findings from this review suggest that enhanced faculty development, updated guidelines incorporating the latest evidence on multimedia design, and fostering a culture of conscientious development of online lectures are all necessary for the continued expansion and application of online education.

**Proposed glossary for online teaching modalities.**

- Traditional lecture
  - Description: delivered live, in-person, and with no or minimal online component; typically limited student-lecturer interaction, unless a flipped classroom format is applied
  - Design components: video, slide decks, and drawing on projector screen or blackboard
  - Interactivity: minimal (students ask questions, but do not influence lecture output or pace)
- Online lecture
  - Description: intended for students to independently watch online, at their own pace; defined by low student interaction with the teaching modality (in some ways akin to a traditional lecture except viewed online)
  - Design components: audio, slide decks, drawings on blackboard (similar to Khan Academy or other educational channels), talking head
  - Interactivity: low to minimal (students can control speed of lecture, rewind, and fast-forward)
- Online module
  - Description: intended for students to independently complete online, at their own pace; involves interactivity, in which students “click through” the module or complete “drag and drop” or other activities
  - Design components: “click-through” modules, embedded exercises (eg, matching, multiple choice questions); may also include components of online lectures
  - Interactivity: moderate to high (students actively engage with the online interface)

### Limitations

The definition of online lecture utilized in this scoping review excluded interactive teaching tools such as self-paced online modules, meaning that it did not comprehensively capture all literature involving the use of online learning modalities in medical education. This was a purposeful decision given our understanding that Richard Mayer’s principles of multimedia design were initially developed through experimentation on traditional slide deck lectures. A final limitation is that although the majority of studies did not describe the use of multimedia design principles, it is possible that these concepts were employed without being explicitly mentioned.

### Conclusion

The integration of online lectures into undergraduate medical education is well-received by students and appears to improve knowledge, clinical skills, and other learning outcomes. Moreover, it appears that the use of multimedia design principles is not yet standard practice in the development of online lectures for medical students. As the adoption of flipped classroom learning and online lectures continues to expand, employing multimedia design principles could further optimize the potential for student learning. Further research on the design of online lectures and other online teaching modalities, enhanced faculty development, incorporation of best practice, and recognition of the importance of conscientious design are critical as online lectures become a mainstay of undergraduate medical education.

## Appendix

Multimedia Appendix 1 Summary of included studies.

## Abbreviations

AAMC-IIME: Association of American Medical Colleges Institute for Improving Medical Education
NOS: Newcastle-Ottawa Scale
OSCE: objective structured clinical examination
USMLE: United States Medical Licensing Examination
